# Nicorandil prevents endothelial dysfunction due to antioxidative effects via normalisation of NADPH oxidase and nitric oxide synthase in streptozotocin diabetic rats

**DOI:** 10.1186/1475-2840-10-105

**Published:** 2011-11-23

**Authors:** Ken-ichi Serizawa, Kenji Yogo, Ken Aizawa, Yoshihito Tashiro, Nobuhiko Ishizuka

**Affiliations:** 1 Product Research Department, Chugai Pharmaceutical Co., Ltd., Gotemba, Shizuoka 412-8513 Japan

**Keywords:** Endothelial dysfunction, Diabetes, Nicorandil, Reactive oxidative species, eNOS, NADPH oxidase

## Abstract

**Background:**

Nicorandil, an anti-angina agent, reportedly improves outcomes even in angina patients with diabetes. However, the precise mechanism underlying the beneficial effect of nicorandil on diabetic patients has not been examined. We investigated the protective effect of nicorandil on endothelial function in diabetic rats because endothelial dysfunction is a major risk factor for cardiovascular disease in diabetes.

**Methods:**

Male Sprague-Dawley rats (6 weeks old) were intraperitoneally injected with streptozotocin (STZ, 40 mg/kg, once a day for 3 days) to induce diabetes. Nicorandil (15 mg/kg/day) and tempol (20 mg/kg/day, superoxide dismutase mimetic) were administered in drinking water for one week, starting 3 weeks after STZ injection. Endothelial function was evaluated by measuring flow-mediated dilation (FMD) in the femoral arteries of anaesthetised rats. Cultured human coronary artery endothelial cells (HCAECs) were treated with high glucose (35.6 mM, 24 h) and reactive oxygen species (ROS) production with or without L-NAME (300 μM), apocynin (100 μM) or nicorandil (100 μM) was measured using fluorescent probes.

**Results:**

Endothelial function as evaluated by FMD was significantly reduced in diabetic as compared with normal rats (diabetes, 9.7 ± 1.4%; normal, 19.5 ± 1.7%; *n *= 6-7). There was a 2.4-fold increase in p47^phox ^expression, a subunit of NADPH oxidase, and a 1.8-fold increase in total eNOS expression in diabetic rat femoral arteries. Nicorandil and tempol significantly improved FMD in diabetic rats (nicorandil, 17.7 ± 2.6%; tempol, 13.3 ± 1.4%; *n *= 6). Nicorandil significantly inhibited the increased expressions of p47^phox ^and total eNOS in diabetic rat femoral arteries. Furthermore, nicorandil significantly inhibited the decreased expression of GTP cyclohydrolase I and the decreased dimer/monomer ratio of eNOS. ROS production in HCAECs was increased by high-glucose treatment, which was prevented by L-NAME and nicorandil suggesting that eNOS itself might serve as a superoxide source under high-glucose conditions and that nicorandil might prevent ROS production from eNOS.

**Conclusions:**

These results suggest that nicorandil improved diabetes-induced endothelial dysfunction through antioxidative effects by inhibiting NADPH oxidase and eNOS uncoupling.

## Background

Diabetes mellitus is regarded as an independent major risk factor for the development of cardiovascular disease, since long-term survival and freedom from cardiac events were reduced in diabetic coronary angioplasty patients [1-3]. Endothelial dysfunction plays a central role in diabetic vascular diseases [4]. A common mechanism underlying this endothelial dysfunction could involve increased production of reactive oxygen species (ROS) in vascular tissue [5]. High glucose greatly increases endothelial superoxide production [6], leading to an eNOS uncoupling state, followed by reduction of NO production and increased ROS production [7-11] which act to quench NO. Reduced NO availability will lead to attenuation of its beneficial vascular effects such as vasodilation, regulation of vascular smooth muscle proliferation, and expression of cellular adhesion molecules involved in the initiation of atherosclerotic plaque formation [12]. Therefore, increased ROS production in diabetes has been speculated to reduce endothelial NO availability, leading to endothelial dysfunction [13,14].

Nicorandil, an anti-angina agent with ATP-sensitive potassium channel opening and nitrate-like activity, reportedly improves prognosis in patients with angina pectoris via preconditioning effects [15], and also exerted endothelial protective effects in both clinical settings and animal studies. Long-term administration of nicorandil significantly improved endothelial function in patients with ischemic heart disease or with cardiovascular risk factors, as evaluated by measurement of flow-mediated dilation (FMD) in forearm arteries [16,17]. In the swine heart, nicorandil reduced myocardial no-reflow after ischemia reperfusion by protecting endothelial function [18]. In human umbilical vein endothelial cells, nicorandil inhibited apoptosis induced by serum starvation by inhibiting ROS production [19]. Furthermore, nicorandil protected from diabetic through inhibition of the production of ROS stimulated by high glucose [20]. Therefore, we hypothesised that nicorandil can prevent diabetic endothelial dysfunction.

In the present study, we investigated the protective effect of nicorandil on endothelial function in streptozotocin (STZ)-induced diabetic rats by measuring FMD in femoral arteries using a high-resolution ultrasound system under *in vivo *conditions in which blood flow, many humoral factors and nerve activity were maintained. The mechanism underlying the protective action of nicorandil was also investigated in relation to ROS production in the endothelium both *in vivo *and *in vitro*.

## Methods

### Animals

Male Sprague-Dawley rats (Charles River Japan, Yokohama, Japan, 6 weeks old, 200-240 g) were used in all experiments. All rats were fed ordinary laboratory chow and allowed free access to water under a constant light and dark cycle of 12 h. Diabetes was induced by intraperitoneal administration of STZ (40 mg/kg) once a day for 3 days. One week after STZ administration, glucose concentrations were measured. Diabetes was considered to have been induced when the glucose level was higher than 250 mg/dL. Nicorandil (15 mg/kg/day) and tempol (20 mg/kg/day) were administered in drinking water for one week, starting 3 weeks after STZ administration.

All animal procedures were conducted in accordance with Chugai Pharmaceutical's ethical guidelines for animal care, and all experimental protocols were approved by the Animal Care Committee of the institution and conformed to the Guide for the Care and Use of Laboratory Animals published by the US National institutes of Health.

### Measurement of FMD

Four weeks after STZ administration, blood pressure (tail-cuff method, BP-98A Softron, Tokyo, Japan) and blood glucose were measured, and the rats were anaesthetised with thiobutabarbital with constant monitoring of rectal temperature. The animals were kept stable with a heated sheet and warming lamps directed at each rat.

Femoral arterial diameter and Doppler flow were measured using a high-resolution ultrasound system (Vevo 770, VisualSonics, Toronto, Canada). The femoral artery was visualised with a 30- or 40-MHz transducer. After identification of the femoral artery by its characteristic flow pattern, the probe position was optimised to show clear vessel wall/lumen interfaces and fixed throughout the investigations. Experiments were started over a 15-min equilibration period and when body core temperature (37 ± 1°C) was stable.

FMD measurement in rats was described previously [21]. Ultrasound diameter and Doppler-flow measurements were obtained from longitudinal sections of the femoral artery before and after 5 min of hindlimb ischemia. Ischemia and reperfusion of the hindlimb were achieved with a snare occluder positioned upstream from the site to be visualised, around the common iliac artery, through a trans-abdominal access. The snare occluder consisted of a 5-0 nylon surgical suture around the artery and passed through a 4 cm PE-200 tube, and the skin was closed with surgical clips. Hindlimb ischemia was achieved by pulling on the filament through the tube and clamping with a clamp.

After an equilibration period, baseline recordings were taken and the common iliac artery was occluded with the snare occluder. Flow arrest was confirmed by abrogation of the Doppler signal. After 5 min of ischemia, the hindlimb was reperfused by release of the occluder. The changes in flow velocity and the diameter of the femoral artery were monitored at 0, 0.5, 1, 2 and 3 min after reperfusion. In this study, FMD was decided as the peak changes of femoral artery diameter measured at 1 min after reperfusion, since clinical guideline for FMD assessment defined the peak changes of brachial artery diameter as an endothelial function, which is occurred about 1 min after reperfusion [22].

For evaluation of endothelium-independent vasodilation, nitroglycerin (NTG) was administered after FMD measurement to the same rats with 10 min breaks between measurements. After obtaining baseline recordings for diameter, NTG (5 μg/kg) was intravenously administered via a jugular vein catheter. Changes in femoral artery in the diameters were monitored at 10, 30, 60 and 120 sec after administration.

### Western blot analysis

Femoral arteries were harvested and frozen in liquid N_2 _immediately after isolation and stored in a -80°C in a freezer until measurement of protein by Western blotting. The femoral artery was homogenised in homogenisation buffer, composed of 25 mM Tris-HCl (pH 7.4), 1 mM dithiolthreitol, 25 mM sodium fluoride, 1 mM sodium orthovanadate, protease inhibitor cocktail tablet, phosphatase inhibitor cocktail and 1% Triton X-100. The homogenates were centrifuged at 14,000 *g *for 20 min at 4°C. Supernatants were collected, and protein concentrations were determined using a BCA Protein Assay Kit (Thermo Scientific, Woltham, USA). Equal amounts of protein extracts were separated on 10% SDS-polyacrylamide gel and immobilised on polyvinylidene difluoride (PVDF) membranes (Millipore, Billerica, USA). The membranes were blocked in PVDF Blocking Reagent (TOYOBO, Osaka, Japan), and incubated with anti-p47^phox ^antibodies, anti-eNOS antibodies (Santa Cruz Biotechnology, Santa Cruz, USA) or anti-GTP cyclohydrolase I (GCH-I) antibodies (Abnova, Taipei, Taiwan). After washing, the membranes were incubated with anti-rabbit or anti-goat IgG conjugated with horseradish peroxidise (Santa Cruz Biotechnology). Immunoreactive signals were visualised with SuperSignal West Dura Extended Duration Substrate (Thermo Scientific), and detected using a ChemiDoc XRS system (Bio-Rad Laboratories, Hercules, USA). Each protein signal was normalised to β-actin expression from the same sample.

### Measurement of serum NOx

For the measurement of NO excretion, the total nitrate and nitrite (NOx) concentrations were measured by the Griess method with a Total Nitric Oxide Assay Kit (Enzo Life Sciences, Farmingdale, USA). Serum samples were diluted 1:2 into reaction buffer and ultra-filtered through a 10,000 molecular weight cut-off filter. Samples were read at 540 nm with a Microplate Reader (Molecular Devices, Sunnyvale, USA).

### Cell culture and treatment

Normal human coronary artery endothelial cells (HCAECs) were purchased from Lonza (Walkersville, MD, USA). HCAECs at passages 3-5 were cultured with EBM-2 supplemented with 5% fetal bovine serum (Lonza). For the measurement of ROS production, the cells were seeded onto plastic dishes (1 × 10^5 ^cells/2 mL/dish) and cultured as monolayers in a 5% CO_2 _humidified incubator at 37°C. After overnight incubation, HCAECs were exposed to normal glucose (5.6 mM) or high glucose (35.6 mM) for 24 h. Nicorandil (100 μM), apocynin (100 μM, Calbiochem, Darmstadt, Germany) or L-NAME (300 μM, St Louis, Sigma-Aldrich) was added over the same period.

### Measurement of ROS production using the fluorescent probe in HCAECs

The ROS production level was monitored using the fluorescent probe 2', 7'-dichlorodihydrofluorescein diacetate (H_2_DCF-DA) (Invitrogen, Carlsbad, USA). Briefly, cultured cells were incubated with 10 μM H_2_DCF-DA for 45 min in a 37°C incubator, and 2', 7'-dichlorofluorescein (DCF) fluorescence was then quantified employing confocal microscopy (Zeiss Axiovert 200) from at least 20 randomly-selected cells/dish, using three dishes for each experimental condition.

### Statistical analyses

All data are expressed as mean ± SE. The *n *values refer to the number of individual animals on which experiments were performed. The statistical significance of differences was determined using Tukey's Multiple Comparison Test. Probability values less than 0.05 were considered significant. Statistical analyses were performed using SAS version 8.2 software (SAS Institute, Cary, USA).

## Results

### Animals

Body weight, blood glucose, blood pressure and heart rate are shown in Table 1. For 4 weeks after STZ administration, body weight increased in each group of rats, although the increase in the STZ rats was smaller than that in normal rats. Blood glucose levels in STZ rats were significantly higher than those in normal rats. Systolic blood pressure was slightly increased while heart rate was significantly decreased in STZ rats. Nicorandil influenced none of these parameters.

**Table 1:** Body weight, blood glucose, blood pressure and heart rate at 4 weeks after STZ administration

	normal	STZ	STZ+nicorandil
N	7	6	6
Body weight (g)	417.8 ± 8.7	299.4 ± 15.4*	327.7 ± 9.8*
Blood glucose (mg/dL)	96.7 ± 1.7	392.3 ± 17.4*	424.5 ± 16.1*
Systolic blood pressure (mmHg)	121.8 ± 1.8	135.4 ± 5.9	133.5 ± 3.2
Heart rate (bpm)	359.4 ± 11.9	312.4 ± 14.5*	334.5 ± 11.4

*p < 0.05 vs normal

### FMD in STZ-induced diabetic rats

**Figure 1:**
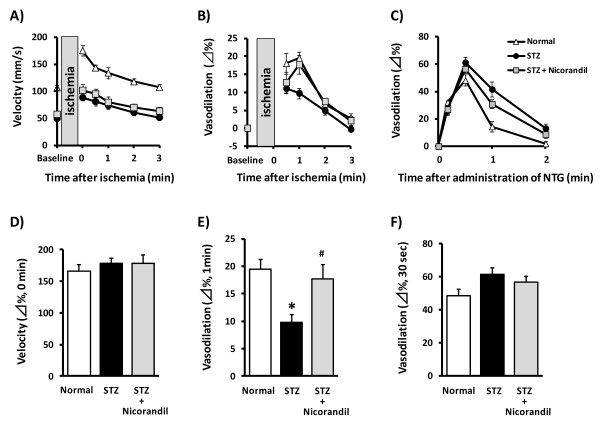
**Effect of nicorandil on STZ-induced endothelial dysfunction in rats**. Ultrasound measurements were performed at 4 weeks after STZ administration. Time courses of velocity (A), FMD (B) and NTG-induced vasodilation (C) in rat femoral arteries were measured after 5-min ischemia. *Δ*% of velocity (D) and NTG-induced vasodilation (F) were similar among groups. FMD peaked at 1 min after reperfusion and then decreased in diabetic rats, showing improvement by nicorandil (E). *p < 0.05 vs normal, #*p *< 0.05 vs STZ (*n *= 6-7).

Endothelial function was evaluated by the measurement of FMD in rat femoral arteries. Reperfusion after 5-min hindlimb ischemia instantaneously increased flow velocity (i.e. reactive hyperaemia) compared with baseline femoral artery flow, followed by a rapid decay to baseline values at around 3 min (Figure 1A). The increase in flow velocity was associated with a delayed increase in femoral arterial vasodilation that peaked at 1 min after reperfusion (Figure 1B). This delayed vasodilation in the femoral artery was observed as FMD. In the evaluation of endothelium-independent vasodilatory potency, intravenous administration of NTG led to vasodilation of the femoral artery that peaked at 30 sec (Figure 1C). In diabetic rats, peak velocity in the femoral artery was lower than that in normal rats (normal, 106.7 ± 5.0; STZ, 49.5 ± 2.5 mm/s; *n *= 6-7, Figure 1A) at baseline (i.e. before ischemia). However, hyperaemia, expressed as the % change in peak velocity just after reperfusion, did not differ between normal and STZ rats (Figure 1D). FMD was decreased in STZ rats compared with normal rats (normal, 19.5 ± 1.7; STZ, 9.7 ± 1.4%; *n *= 6-7, Figure 1B, E). NTG-induced vasodilation was similar in normal and STZ rats (Figure 1C, F).

For the evaluation of nicorandil effects on endothelial function in STZ rats, nicorandil (15 mg/kg/day) was administered in drinking water for one week starting 3 weeks after STZ administration. Nicorandil restored the reduced FMD in STZ rats to almost same level as in normal rats (STZ + Nicorandil, 17.7 ± 2.6%; *n *= 6, Figure 1B, E), whereas nicorandil affected neither NTG-induced vasodilation nor flow velocity. In normal rats, nicorandil showed no significant changes in FMD (normal, 18.8 ± 4.0; nicorandil, 20.9 ± 1.7%; *n *= 5-6). These results demonstrated nicorandil ameliorate endothelial dysfunction, without changing flow velocity or vascular smooth muscle function, in STZ rats.

### Involvement of oxidative stress in decreased FMD in STZ-induced diabetic rats

**Figure 2:**
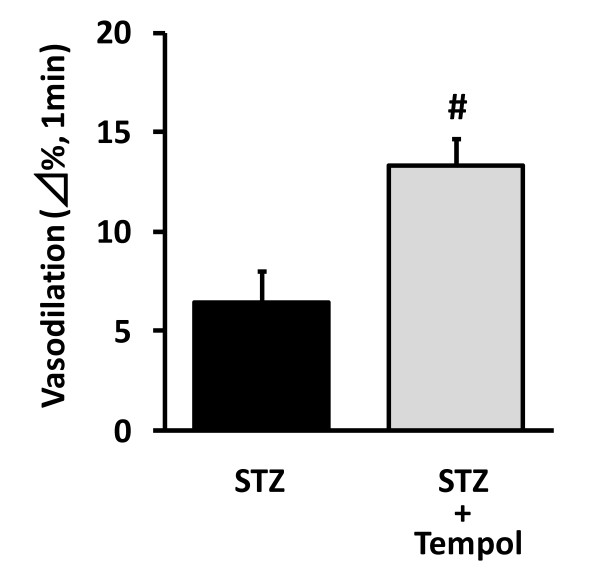
**Effect of tempol on STZ-induced endothelial dysfunction in rats**. Ultrasound measurements were performed at 4 weeks after STZ administration. Data indicate the % changes of femoral diameter at 1 min after reperfusion. Tempol improved the FMD decrease in diabetic rats. #*p *< 0.05 vs STZ (*n *= 6).

**Figure 3:**
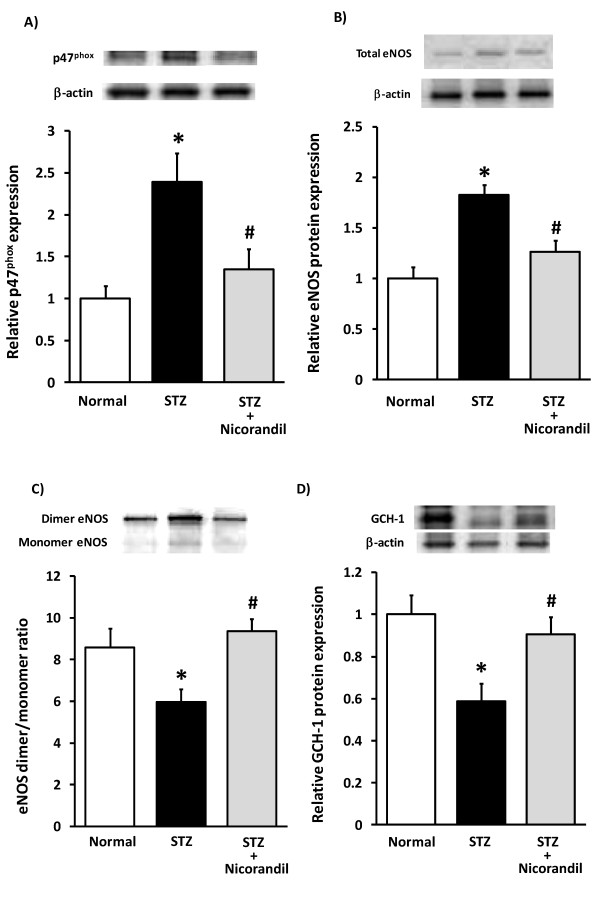
**Effects of nicorandil on p47^phox ^and total eNOS, GCH-1 expressions and eNOS uncoupling in rat femoral artery**. Nicorandil normalised increased p47^phox ^protein (A) and total eNOS protein (B) in diabetic rat femoral arteries. Nicorandil also normalized decreased eNOS dimer/monomer ratio (C) and GCH-1 protein (D) in diabetic rat femoral arteries. Femoral arteries were harvested 4 weeks after STZ administration. *p < 0.05 vs normal, #*p *< 0.05 vs STZ (*n *= 6-8).

To clarify the involvement of oxidative stress in the endothelial dysfunction in STZ rats, the effect of tempol was investigated. Tempol significantly reversed the FMD decrease in STZ rats (STZ, 6.5 ± 1.5; STZ + Tempol, 13.3 ± 1.4%; *n *= 6, Figure 2). Because NADPH oxidase is known to be a potential source of vascular superoxide production, we measured its expression in STZ rat femoral arteries. In STZ rats, p47^phox ^protein, which is major NADPH oxidase component, was increased in the femoral artery (Figure 3A). To examine whether endothelial dysfunction is associated with reduced eNOS expression, we measured total eNOS expression in the rat femoral artery. Total eNOS expression was increased in STZ rat femoral arteries (Figure 3B). However, this increase in total eNOS expression did not raise serum NOx levels in STZ rats and nicorandil also showed no significant changes (normal, 9.3 ± 0.6; STZ, 11.3 ± 1.8; STZ + nicorandil, 13.2 ± 3.0 μM, *n *= 6-8). In oxidative state, reduction in tetrahydrobiopterin (BH_4_) results in uncoupling of eNOS, resulting in production of superoxide by eNOS monomer [8]. In this study, eNOS dimer/monomer ratio was decreased (Figure 3C), and GCH-I expression, a synthetase of BH_4_, was also decreased in STZ rat femoral arteries (Figure 3D). Nicorandil restored p47^phox^, GCH-1 and total eNOS expressions and also eNOS dimer/monomer ratio in STZ rat femoral artery to normal values (Figure 3).

### High glucose-induced ROS production in HCAECs

**Figure 4:**
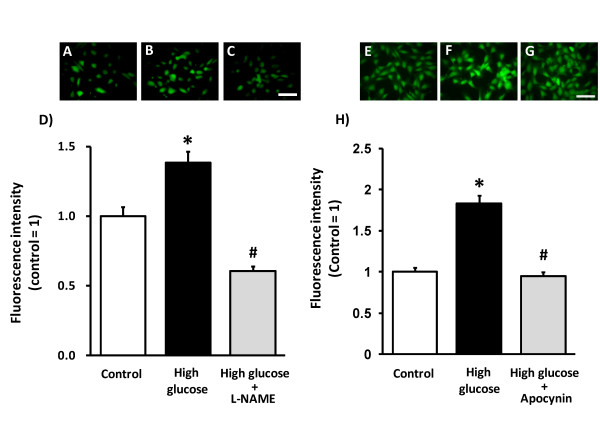
**Effect of L-NAME and apocynin on ROS production induced by high-glucose treatment in HCAECs**. HCAECs were exposed to high glucose (35.6 mM) for 24 h, and L-NAME (300 μM) or apocynin (100 μM) for the same period. Representative images show DCF fluorescence in control (A, E), high-glucose treatment (B, F) and high-glucose + L-NAME (C) or + apocynin (G). High-glucose treatment increased ROS production, which was prevented by L-NAME (D) or apocynin (H). *p < 0.05 vs control, #*p *< 0.05 vs high-glucose (20 cells/dish from 2-3 dishes). Scale bar = 100 μm.

**Figure 5:**
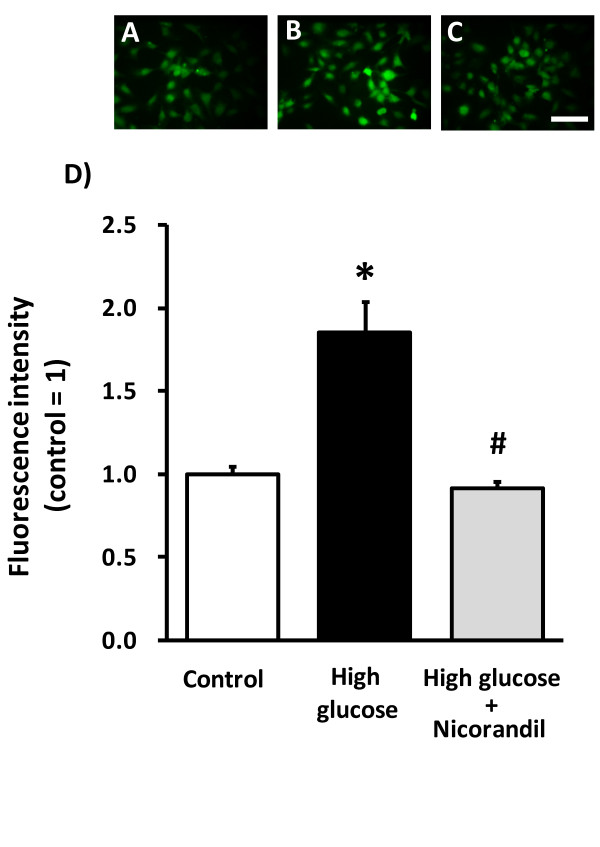
**Effect of nicorandil on ROS production induced by high-glucose treatment in HCAECs**. HCAECs were exposed to high glucose (35.6 mM) for 24 h, and nicorandil (100 μM) for the same period. Representative images show DCF fluorescence in control (A), high-glucose treatment (B) and high-glucose + nicorandil (C). High-glucose treatment increased ROS production, which was prevented by nicorandil (D). *p < 0.05 vs control, #*p *< 0.05 vs high-glucose (20 cells/dish from 3 dishes). Scale bar = 100 μm.

To clarify the possibility that eNOS itself might serve as a source of superoxide in the diabetic state, we examined the influence of the NOS inhibitor L-NAME on high glucose-induced ROS production in HCAECs. High glucose increased DCF fluorescence in HCAECs. Interestingly, L-NAME decreased high glucose-induced ROS production in HCAECs (Figure 4A-D), indicating that eNOS to be an important superoxide source. Furthermore, apocynin, an NADPH oxidase inhibitor, also decreased high glucose-induced ROS production in HCAECs (Figure 4E-H). Nicorandil also prevented high glucose-induced ROS production in HCAECs (Figure 5A-D).

## Discussion

The present study demonstrated that nicorandil protected from the endothelial dysfunction assessed by the FMD reduction without affecting endothelium-independent vasodilation in STZ rats. Since earlier report has shown that diabetes-induced endothelial dysfunction was caused by increased ROS [5], there would be 2 possibilities as the underlying mechanisms of endothelial protective effect; Nicorandil could inhibit both the expression and activity of NADPH oxidase, a major source of ROS [23,24], leading to the reduction of ROS production. Secondly, since monomer state of eNOS is a source of ROS but not NO depending on the BH_4 _level [8], nicorandil could inhibit the monomerization of eNOS by increased expression of BH_4 _synthase in arteries of STZ rats, resulting in the reduction of ROS production. The present results suggested that nicorandil may offer a novel strategy for diabetes complication therapy targeting endothelial dysfunction due to ROS production.

### Endothelial dysfunction and NADPH oxidase in diabetes

Endothelial dysfunction is attributable to endothelial ROS production derived from vascular NADPH oxidase, an important vascular source of superoxide [23,24]. Present results are with general agreement with earlier reports that endothelial dependent relaxation of artery was impaired by the increased ROS production via NADPH oxidase in both type I [25,26] and type II diabetic model [27,28]. The present study demonstrated that tempol, a radical scavenger improved the reduced FMD (Figure 2), and that the expression of NADPH oxidase subunit p47^phox ^was increased in femoral artery (Figure 3A) as well as apocynin significantly decreased high glucose-induced ROS production in HCAECs (Figure 4H), indicating that the endothelial dysfunction in STZ rats was caused by the increased oxidative stress produced by NADPH oxidase. Moreover, nicorandil inhibited expression of NADPH oxidase in STZ rat femoral arteries (Figure 3A), and also prevented high glucose-induced ROS production in HCAECs (Figure 5). These results suggest that nicorandil ameliorates endothelial dysfunction by inhibiting ROS production through decreased NADPH oxidase expression in STZ rats. This is in general agreement with a previous report showing that nicorandil can inhibit ROS production through NADPH oxidase activation in response to hypoxia-reoxygenation in human umbilical vein endothelial cells [29].

The increased NADPH oxidase-mediated ROS production in diabetic vessels is at least partially mediated by protein kinase C (PKC) and NF-κB [30,31]. Because nicorandil exerts inhibitory effects on PKC activity [32] and NF-κB activation [33], nicorandil might prevent the NADPH oxidase increase in diabetic rats by inhibiting PKC or NF-κB. Moreover, it has been suggested that mitochondrial ROS may activate vascular NADPH oxidase through a PKC-dependent process [34]. Eguchi *et al *suggested that nicorandil inhibits ROS-induced ROS release in the mitochondria of endothelial cells [35]. Therefore, nicorandil may inhibit NADPH oxidase by protecting mitochondria.

### Contribution of eNOS to ROS production in diabetes

eNOS can produce NO or superoxide. In oxidative states, reduction in BH_4_, an essential cofactor of NOS, results in uncoupling of eNOS [8], resulting in production of superoxide by the eNOS monomer whereas the dimer, in the presence of abundant BH_4_, produces mainly NO [14]. In the diabetic state, eNOS is believed to serve as another source of ROS [25,36]; high glucose can induce an eNOS uncoupling state due to BH_4 _deficiency [6], leading to reduced NO production and increased superoxide production [25,37]. In fact, there are clinical and experimental reports describing administration of BH_4 _as ameliorating endothelial dysfunction [38,39]. In the present study, the expression of GCH-1, a major enzyme for BH_4 _synthesis, was decreased in STZ rats (Figure 3D), which was compatible with the decrease in eNOS dimer/monomer ratio (Figure 3C). These results suggest that ROS production in STZ rats could be derived from un-coupled state of eNOS. In fact, high glucose-induced ROS production in HCAECs was completely inhibited by the NOS inhibitor L-NAME (Figure 4D), suggesting that ROS production in STZ rats could mainly be derived from eNOS.

Nicorandil was known to increase the eNOS expression and eNOS activity in several studies; not only in normal rat hearts [40] but in several disease models such as salt-sensitive Dahl rats [41,42], myocardial infarcted rats [43] and monocrotaline-induced pulmonary hypertensive rats [44], nicorandil increased eNOS expression, which was inhibited by glybenclamide [40]. Increased NO release by nicorandil was correlated with enhancement of eNOS phosphorylation in cultured rat cardiac fibroblasts [45]. These increases in eNOS expression were related to the improvement of symptoms. On the other hand, the present study demonstrated that nicorandil decreased the increased expression of total eNOS in STZ rats (Figure 3B) with improvement of endothelial function. To our knowledge, this is the first demonstration that nicorandil inhibits the increased expression of eNOS in STZ-induced diabetic rats. There is a possibility about the mechanism of inhibition of eNOS expression by nicorandil. In diabetes rats, hyperglycemia increased superoxide production through activation of the mitochondrial electron transport chain [46], which activated PKC, resulting in eNOS upregulation in endothelium [4,25]. Because nicorandil and diazoxide have been known to reduce the mitochondrial ROS production [47], the reduction of the mitochondrial ROS production by nicorandil may be responsible for the inhibition of increased expression of eNOS in STZ rats. Further investigation is required to clarify the exact mechanisms by which nicorandil regulates eNOS expression.

### Action mode of nicorandil

Since nicorandil is known to have K_ATP _channel opening effect and nitrate like activity, it is of interest which action contributed to the endothelial protection in this study. Earlier reports indicate the importance of K_ATP _channel in the endothelial protection by nicorandil; nicorandil but not isosorbide dinitrate (ISDN) improved FMD in the patients with ischemic heart disease [16]. We previously reported that nicorandil and diazoxide but not ISDN improved FMD in paclitaxel-treated rats [48]. These results suggest the importance of K_ATP _channel in the endothelial protection by nicorandil although we could not rule out the contribution of nitrate like activity. Further investigation will be required to clarify the action mode of nicorandil on diabetes-induced endothelial dysfunction.

### Protective effect of tempol from endothelial dysfunction

Tempol, a cell permeable superoxide dismutase mimetic, protects animals and mammalian cells from cytotoxicity induced by oxygen-free radicals like hydroxyl radicals, H_2_O_2_, and O_2_^- ^[49]. An additional property of tempol as antioxidant is that it can penetrate cell membranes, and hence react with both intracellular and extracellular oxygen-free radicals. These properties make tempol attractive for treatment of cardiovascular disease associated with oxidative stress (e.g. diabetes, hypertension) [50]. In fact, tempol can restore endothelium-dependent relaxation in isolated small artery from rats with type I and type II diabetes through the reduction the levels of reactive oxygen species [51]. In the present study, chronic administration of tempol inhibited the endothelial dysfunction. Recently, much higher dose of tempol (100 mg/kg/day in drinking water, 12 days) than that in the present study has no effect on the body weight, blood glucose and blood pressure in STZ rat [52], although same higher dose of tempol has hypotensive effects in hypertensive rats [50]. Taken together, the present result suggests that tempol, as well as nicorandil, may be another candidate for treatment of diabetic endothelial dysfunction.

### Usefulness of FMD measurement in rats

Herein, we clearly observed reduced FMD in STZ rats without changes in NTG-induced endothelium independent relaxation (Figure 1). These results are consistent with those in earlier reports showing that acetylcholine-induced relaxation was diminished without affecting endothelium-independent relaxation in isolated vascular beds from STZ rats [27,38,53]. These results raise the possibility that FMD measurement will be a useful method for investigating endothelial dysfunction in diabetic rats.

We consider evaluation of endothelial function by measuring FMD in live rats to have at least 3 potential advantages. First, FMD measurement can evaluate endothelial function under both physiological and pathological conditions. In the diabetic state, plasma levels of humoral factors, autonomic nervous activity and regional blood flow are altered. Hyperglycaemia increases the levels of ROS [54], oxidative LDL [55] and endothelin [56], thereby impairing endothelial function in diabetic rats. These lines of evidence indicate that results from isolated arterial strips may underestimate the precise *in vivo *situation of endothelial dysfunction because these humoral factors are absent from experiments on isolated tissues. Second, we consider rats to be more useful than large animals in many situations, although FMD measurement can be applied not only for clinical diagnosis but also experiments in large animals such as dogs [57] and pigs [58]. We can conduct rat experiments using small amounts of drugs, due to the animal's low body weight. Furthermore, many rat disease models are available. Finally, FMD makes it possible to repeatedly measure endothelial function in individual rats. Time-dependent changes in pathological conditions or chronic effects of drugs on endothelial function can be traced in the same rat by permanent implantation of an iliac artery occluder. Therefore, in addition to the classical endothelial function measurement method in isolated arteries, measurement of FMD in living rats should also provide useful information for not only elucidating endothelial physiology and pathology but also ascertaining the roles of endothelial protective agents.

### Clinical significance

In diabetic patients, morbidity and mortality rise due to diabetic vascular complications such as atherosclerosis, retinopathy and nephropathy, triggered by endothelial dysfunction. Glycaemic control agents, statins and renin-angiotensin system inhibitors ameliorate endothelial dysfunction [59], thereby improving prognosis. In this study of diabetic rats, nicorandil protected against endothelial dysfunction through inhibition of ROS production without glycaemic control. This result may suggest that nicorandil can improve the prognosis of patients with ischemic heart disease and diabetic complications through not only the pharmacological preconditioning effect, but also an endothelial protective effect. In fact, the IONA [60] and J-CAD [61] studies demonstrated nicorandil to improve the prognosis of patients with ischemic heart disease and diabetes.

## Conclusions

The present study demonstrated that nicorandil ameliorated endothelial dysfunction in STZ rats through an antioxidative effect exerted by normalising p47^phox ^and eNOS uncoupling.

## Competing interests

The authors declare that they have no competing interests.

## Authors' contributions

KS carried out the animal and molecular studies and drafted the manuscript. KY and YT carried out the animal studies and helped to draft the manuscript. AK carried out experiments using cultured cells. NI conceived of the study, and participated in its design and coordination and helped to draft the manuscript. All authors read and approved the final manuscript.
